# Supplementary material for: Intracellular bound chlorophyll residues identify 1 Gyr-old fossils as eukaryotic algae
Source: Nat Commun. 2022 Jan 10;13:146. doi: 10.1038/s41467-021-27810-7 (PMC8748435; doi:10.1038/s41467-021-27810-7)
Supplement: Supplementary file 1 — Supplementary Information [file 41467_2021_27810_MOESM1_ESM.pdf]

Supplementary Information for

Intracellular bound chlorophyll residues identify 1 Gyr-old fossils as eukaryotic algae

Sforna Marie Catherine[1,*], Loron Corentin C.[1], Demoulin Catherine F.[1], François Camille[1,2], Cornet Yohan[1], Lara Yannick J.[1], Grolimund Daniel[3], Ferreira Sanchez Dario[3], Medjoubi Kadda[4], Somogyi Andrea[4], Addad Ahmed[5], Fadel Alexandre[5], Compère Philippe[6], Baudet Daniel[7], Brocks Jochen J.[8] & Javaux Emmanuelle J.[1,*]

[1]*Early Life Traces & Evolution-Astrobiology, UR Astrobiology, University of Liège, Liège, Belgium*

[2]*Commission for the Geological Map of the World, Paris, France*

[3]*Paul Scherrer Institut, Swiss Light Source, CH-5232 Villigen PSI, Switzerland*

[4]*Synchrotron Soleil, Saint-Aubin – BP 48, France*

[5]*Unité Matériaux et Transformations (UMR CNRS 8207), Université Lille 1 - Sciences et Technologies, Villeneuve d'Ascq, France*

[6]*Functional and Evolutive Morphology, Department of Biology, Ecology and Evolution, UR FOCUS, and Center for Applied Research and Education in Microscopy (CAREM-ULiège), University of Liège, Liège, Belgium*

[7]*Geodynamics & Mineral Resources Service, Royal Museum for Central Africa, Belgium*

[8]*Research School of Earth Sciences, The Australian National University, Canberra, ACT, Australia*

*Corresponding authors: mcsforna@uliege.be, ej.javaux@uliege.be

This supplementary information contains Supplementary text, 6 Supplementary Tables, 20 Supplementary Figures and 44 references.

Geological settings

The studied microfossils come from a pristine drill core (Kanshi SB13) carried out in the Congo Basin (Mbuji-Mayi Supergroup, Supplementary Fig. 1) during the 1950's and stored at the Royal Museum for Central Africa (Geodynamic and Mineral Resource Service, Tervuren, Belgium). Covering Angola, Democratic Republic of the Congo, Central African Republic and Republic of the Congo, the Congo Basin is an intracratonic basin recovering up to 9 km of sediments deposited between the Mesoproterozoic and the Neogene[1-3]. The Mbuji-Mayi Supergroup was deposited in the Sankuru-Mbuji-Mayi-Lomami-Lovoy (SMLL) basin, a failed-rift basin[1] in the northern part of the Congo Basin (DRC, Supplementary Fig. 1). The Mbuji-Mayi Supergroup was deposited in shallow marine to evaporitic marine and lacustrine environments[4]. It is mostly unaffected by regional metamorphism[5] with maximum temperature of burial ~200°C [6]. A detailed petrological description of the investigated drill core is provided by (7) and (5, 8), and revised by (9, 10). The Mbuji-Mayi Supergroup consists of two groups. The lower group, BI Group, ~500 m thick, is a siliciclastic sequence dated between ca. 1065 to 1030 Ma by U-Pb dating on diagenetic monazites and xenotimes[11] and < 1174 ± 22 Ma by U-Pb dating on detrital zircons[10]. The top group, BII Group, ~1000 m thick, is a badly constrained-in-time upper carbonated sequence intercalated with sparse organic-rich shales. It is dated younger than 950 Ma by K-Ar method[12,13] and based on C, O, and Sr isotopic correlations it is younger than 800 Ma[14].

The Kanshi SB13 drill core samples 350 m of the BII group[15] and shows horizons displaying a large diversity of exquisitely well-preserved organic-walled microfossils[15]. Forty-nine taxa were reported, comprising 11 unambiguous eukaryotes, 10 possible eukaryotes and 28 probable prokaryotes[15]. The investigated microfossils come more especially from the BIIc6 formation, showing the higher total species richness of the drill core[16], and in particular at 123 m depth where *A. tetragonala* specimens were retrieved (KN22 and KN23). The BIIc6 formation consists of 27 m of grey stromatolitic shaly dolomites intercalated with dark shales horizons. The BIIc6 stromatolites grew in relatively shallow-water with little detrital fluxes while shale beds correspond to installment of dysoxic/anoxic conditions linked with sea-level variations[16].

Petrography of the mineral matrix

The petrographical study of the mineral matrix of KN22 and KN23 shales shows that the matrix consists mostly of quartz and clays with presence of K-felspath, calcite, anatase, and rare sulfides (<2%), which are mostly marcasite. Amorphous organic matter is found mostly associated to marcasite. Previous study led in the Early Life laboratory has shown that the clays are a mixture of illite-micas, chlorite, kaolinite and 10-14 mixed layer clays[16]. The SR-µXRF mapping (Supplementary Fig. 20) confirms this petrological analysis, as shown by the distribution of Fe and K. The distribution of Ti highlights the presence of anatase, which also contains some V. The Ca map shows the limited carbonate distribution. Sulfides contain low amounts of Ni, Cu, As and Cr.

Archaeaplastida affinity of A. tetragonala

Our reinvestigation of *A. tetragonala* microfossils evidences that *A. tetragonala* was a siphonocladous filamentous organism, dichotomously branched with equal-diameter branches. The node cell is generally larger than the other cells with a roughly trapezoidal form with two small protuberances to which the branches are attached (Fig. 1). In some specimens, the three branches are still attached (Fig. 1c, 1f), suggesting the nodal cell is not a holdfast structure. *A. tetragonala* thus corresponds to fragments of a larger organism which could have been either a simple-branched organism or a heterotrichous organism (with a prostrate section and upright sections). However, evidencing a benthic or pelagic habit is not possible in absence of attachment structure (holdfast) or preservation in place in, or on, the substrate. The presence of tetrapyrrole moieties deriving from chlorophyll within *A. tetragonala* ICIs clearly demonstrates that it was capable of phototrophy and could not be a fungus (obligatory heterotroph) as previously proposed[17]. Photosynthesis is widespread among eukaryotes[18] and is found within the supergroups Excavata (Euglenozoans), TSAR (Alveolata and Stramenopiles) and Archaeaplastida (Rhodophyta, Chlorophyta and Glaucophyta). Unicellular algae within the eukaryotic supergroups can be ruled out based on the multicellularity of *A. tetragonala*. Among multicellular filamentous branching algae, Xanthophytes and Phaeophyceans (Stramenopiles) can also be excluded as *A. tetragonala* specimens do not display dendroid

branching, tissue-grade organization, apical septa suggesting apical growth, or typical reproductive structures[18,19]. Therefore, we assign *A. tetragonala* to the total group Archaeaplastida, where several clades of modern green algae and some florideophyte red algae are known to display a siphonocladous body plan. No distinctive characters of these algae can be found with certainty in *A. tetragonala*. As such, it is possible that *A. tetragonala* represents an extinct stem lineage within Archaeplastida and further taxonomic recognition is puzzling. However, the absence of pit plugs, although difficult to observe in fossils, of multiseriate sections in filaments, of longitudinal division, and the fact that the siphonocladous body plan is a derived character in red algae seems to preclude a rhodophycean affinity[20].

Supplementary Table 1. Reported occurrences of *A. tetragonala* from literature and ages of the fossiliferous formations

Units	Era	References	Ages (Ma)	References
Owk Shale Fm, Kurnoll Group (India)	Neoproterozoic	Shukla et al., 2019 (21)	<1140<br>>500 - <980<br>[disputed]	Crawford and Compston, 1973 (22)<br>Raman and Murty, 1997 (23)
Qiaotou & Changlingzi fms (China)	Neoproterozoic	Xing et al, 1985 (24)	ca. 920-980	Zhang et al, 2016 (25)
Tumen Group (China)	Neoproterozoic	Li et al., 2019 (26) ;<br>Han et al, 2021 (27)	~1000 – 720	See (27) for discussion
Miroedikha Fm (Russia)	Neoproterozoic	Timofeev et al., 1976 (28); Hermann, 1990 (29); Hermann and Podkovyrov, 2008 (17)	~1000-800	Hermann, 1990 (29)
Uk Fm, Southern Urals (Russia)	Neoproterozoic	Jankauskas, 1982 (30); Jankauskas et al., 1989 (31); Hermann and Podkovyrov, 2008 (17)	664±11 - 669±16	Zaitseva et al., 2008 (32)
Zilmerdak Fm, Southern Urals (Russia)	Neoproterozoic	Jankauskas, 1982 (30); Jankauskas et al., 1989 (31); Hermann and Podkovyrov, 2008 (17)	<1030 - 618±13	Zaitseva et al., 2008 (32)
Kwahu Group (Ghana)	late Mesoproterozoic-Neoproterozoic	Couëffé and Vecoli, 2011 (33)	<1115±23- 509±68	Kalsbeek et al., 2008 (34)
Bylot Supergroup (Canada)	late Mesoproterozoic-early Neoproterozoic	Hofmann and Jackson, 1994 (35)	1047	Gibson et al., 2017 (36)
lower Shaler Supergroup (Canada)	late Mesoproterozoic-early Neoproterozoic	Loron et al., 2019 (37)	892±13 - 1232±15	van Acken et al., 2013 (38)<br>Rayner and Rainbird, 2013 (39)
Mbuji-Mayi Supergroup (Democratic Republic of Congo)	late Mesoproterozoic-early Neoproterozoic	Maithy, 1975 (40);<br>Baludikay et al., 2016 (15)	1030-950	François et al., 2017 (11)<br>Cahen et al, 1974; 1984 (12,13)
Atar/El Mreïti Group (Mauritania)	late Mesoproterozoic-early Neoproterozoic	Beghin et al., 2017 (41)	1107±12-1109±22	Rooney et al., 2010 (42)

Supplementary Table 2. Positions of G- and D1-bands (ω-G, ω-D1), Full Width at Half Maximum of G- and D1-bands (FWHM-G, FWHM-D1), Raman reflectance (Rmc0%) and calculated temperatures on ICIs and walls.

	**ω-G (cm^{-1})**	**FWHM-G (cm^{-1})**	**ω-D1 (cm-1)**	**FWHM-D1 (cm-1)**	**Rmc0 %**	**Temperature (°C)**
**ICI**	1594	56	1341	115	2.40	206
**ICI**	1593	52	1341	107	2.45	208
**ICI**	1594	53	1344	110	2.32	203
**ICI**	1594	55	1340	112	2.52	210
**ICI**	1594	52	1341	116	2.48	209
**ICI**	1595	51	1347	117	2.21	199
**ICI**	1595	51	1346	113	2.28	202
**ICI**	1596	50	1349	110	2.13	197
**ICI**	1596	49	1349	110	2.17	198
**Mean**	1595	52	1344	112	2.33	203
**σ**	1	2	3	3	0.13	5
**Walls**	1597	48	1348	115	2.27	201
**Walls**	1598	50	1346	115	2.40	206
**Walls**	1595	54	1343	112	2.42	207
**Walls**	1596	57	1342	120	2.53	210
**Walls**	1593	60	1343	122	2.36	205
**Walls**	1599	52	1343	110	2.63	213
**Walls**	1598	53	1343	117	2.61	213
**Walls**	1595	58	1345	116	2.35	204
**Mean**	**1596**	**54**	**1344**	**116**	**2.45**	**208**
**σ**	**2**	**4**	**2**	**4**	**0.13**	**4**

Supplementary Table 3. Peak areas (in counts per s) for Fe, Ni, Cu and Zn estimated from peak fitting of selected regions (ICI and Wall) from the SR-µXRF maps and enrichment rate calculated between the wall and the ICIs of 9 specimens of *A. tetragonala*.

		A_{Fe}	A_{Ni}	A_{Cu}	A_{Zn}	A_{Ni}/A_{Fe}	A_{Cu}/A_{Fe}	A_{Zn}/A_{Fe}
**Arcta Fig.1 a-f**	**ICI**	$6.74.10^{8}$	$4.27.10^{8}$	$9.69.10^{7}$	$1.49.10^{8}$	0.634	0.144	0.221
	**Wall**	$5.22.10^{8}$	$8.33.10^{7}$	$1.05.10^{8}$	$1.13.10^{8}$	0.160	0.201	0.216
	**ICI/Wall**	*1.29*	*5.13*	*0.923*	*1.319*	***3.96***	*0.71*	*1.02*
**Arcta Fig.1 g-j**	**ICI**	$1.20.10^{9}$	$3.44.10^{8}$	$3.01.10^{8}$	$4.68.10^{7}$	0.287	0.0251	0.039
	**Wall**	$5.99.10^{8}$	$6.01.10^{7}$	$1.76.10^{8}$	$1.06.10^{8}$	0.100	0.294	0.177
	**ICI/Wall**	*2.00*	*5.72*	*1.71*	*0.442*	***2.857***	*0.854*	*0.220*
**Arcta Suppl. Fig. 6**	**ICI**	$1.70.10^{7}$	$1.37.10^{7}$	$5.36.10^{6}$	$3.40.10^{5}$	0.806	0.315	0.0200
	**Wall**	$1.01.10^{7}$	$6.72.10^{5}$	$4.16.10^{6}$	$6.14.10^{5}$	0.0665	0.412	0.0608
	**ICI/Wall**	*1.68*	*20.4*	*1.29*	*0.554*	***12.1***	*0.765*	*0.329*
**Arcta Suppl. Fig. 7**	**ICI**	$4.71.10^{7}$	$7.06.10^{6}$	$1.91.10^{6}$	$4.33.10^{5}$	0.150	0.0406	0.00919
	**Wall**	$4.76.10^{7}$	$1.41.10^{6}$	$4.32.10^{6}$	$8.97.10^{5}$	0.0296	0.0908	0.0188
	**ICI/Wall**	*0.989*	*5.01*	*0.442*	*0.483*	***5.07***	*0.447*	*0.489*
**Arcta Suppl. Fig. 8**	**ICI**	$3.84.10^{6}$	$4.51.10^{6}$	$2.67.10^{5}$	$4.59.10^{4}$	1.17	0.070	0.0120
	**Wall**	$1.45.10^{8}$	$9.79.10^{6}$	$2.07.10^{7}$	$4.98.10^{6}$	0.0675	0.143	0.0343
	**ICI/Wall**	*0.0264*	*0.46*	*0.0129*	*0.00922*	***17.3***	*0.490*	*0.350*
**Arcta Suppl. Fig.11**	**ICI**	$7.04.10^{7}$	$8.33.10^{6}$	$3.91.10^{6}$	$1.31.10^{6}$	0.118	0.056	0.0186
	**Wall**	$7.64.10^{7}$	$2.29.10^{6}$	$3.16.10^{6}$	$1.43.10^{6}$	0.0300	0.0414	0.0187
	**ICI/Wall**	*0.921*	*3.64*	*1.24*	*0.916*	***3.93***	*1.35*	*0.995*
**Arcta Suppl. Fig.12 E-H**	**ICI**	$5.04.10^{8}$	$2.99.10^{8}$	$3.70.10^{8}$	$1.34.10^{7}$	0.598	0.740	0.047
	**Wall**	$2.28.10^{8}$	$9.09.10^{7}$	$3.81.10^{8}$	$2.56.10^{7}$	0.399	1.671	0.112
	**ICI/Wall**	*2.193*	*3.29*	*0.971*	*0.914*	***1.5***	*0.443*	*0.417*
**Arcta Suppl. Fig.13**	**ICI**	$3.41.10^{9}$	$1.57.10^{9}$	$5.71.10^{4}$	$2.28.10^{8}$	0.460	0.002	0.067
	**Wall**	$7.64.10^{7}$	$2.29.10^{6}$	$3.16.10^{6}$	$1.43.10^{6}$	0.0300	0.0414	0.0187
	**ICI/Wall**	*11.18*	*39.35*	*0.0001*	*7.31*	***1.905***	*0.425*	*0.144*
**Arcta Suppl. Fig.14**	**ICI**	$8.94.10^{8}$	$7.54.10^{8}$	$5.60.10^{8}$	$2.09.10^{7}$	0.843	0.626	0.023
	**Wall**	$3.00.10^{8}$	$8.19.10^{7}$	$5.06.10^{8}$	$2.65.10^{7}$	0.273	1.687	0.088
	**ICI/Walll**	*2.98*	*9.206*	*1.107*	*0.789*	***3.089***	*0.443*	*0.417*

Supplementary Table 4. Linear combination fitting (LCF) results for Ni K-edge XANES spectra.

Analysis point	NiOEP	NiTPP	Asphaltene	NiO	$Ni(OH)_2$	NiCO3	$NiSO_4(H_2O)_{6-7}$	Sum	R Factor	Total porphyrinic species ratio
**Fig3 Point 1**	2.3 %	39.0 %	48.6%	3%	0 %	0 %	0 %	92.9 %	0.0017	**96.8%**
**Fig3 Point 2**	3.6 %	45.7 %	47.4 %	7.0 %	0 %	0 %	0 %	103.7 %	0.0024	**93.2 %**
**Fig3 Point 3**	0 %	31.4 %	46.6 %	22.3 %	0 %	0 %	3.2 %	103.6 %	0.0010	**75.3 %**
**Fig3 Point 4**	20.5 %	20.5 %	51.2 %	9.5 %	0 %	0 %	0 %	101.8 %	0.0028	**92.2 %**
**FigS18 Point 1**	46.5	19.1	40.5	0 %	0 %	0 %	4.2 %	110.3	0.0009	**96.2 %**
**FigS18 Point 2**	24.7 %	36.6 %	48.1 %	0 %	0 %	0 %	0 %	109.4	0.0023	**100 %**
**FigS18 Point 3**	4.8 %	54.9 %	40.9 %	0 %	0 %	0 %	0 %	100.6	0.0039	**100 %**

Supplementary Table 5. Chlorophyll and heme concentrations for different phototrophic eukaryotes and yeasts from literature.

**Species**	***Thalassiosira weissflogii***	***Thalassiosira oceanica***	***Emiliana huxleyi***	***Dunaliella tertiolecta***	***Dunaliella salina***	***Scenedesmus dimorphus***	***Saccharomyces cerevisiae***	***Saccharomyces cerevisiae***
**Type**	Diatoms	Diatoms	Coccolithophores	Green algae	Green algae	Green algae	Yeast	Yeast
**Chlorophyll per cell (mM)**	2.55	5.4	12	17	14.5	1.6	N/A	N/A
**Heme per cell (µM)**	12	17	31	61	*36.2-72.5*	*4-8*	0.54-5	0.25
**References**	Gledhill, 2007 (27)	Gledhill, 2007 (27)	Gledhill, 2007 (27)	Gledhill, 2007 (27)	Xu et al., 2016 (28)	Fereirra et al., 2015 (29)	Hanna et al., 2018 (23)	Hanna et al., 2017 (24) Donegan et al., 2019 (25)

Heme values in italic were estimated using the range of chl/heme ratios reported in Gledhill, 2007 (27)

Supplementary Table 6. Parameters and results for tetrapyrrole molar concentration calculations.

**Cells**	**Length** ***L*** **(µm)**	**Measured Radius** ***Rm*** **(µm)**	**Corrected Radius** ^{a} ***Rc*** **(µm)**	**Volume** ^{b} ***Vcell*** **(dm^3)**	**Projected area of the cell** ^{c} A_P **(cm^2)**	**Mean count rate (counts s^{-1})**	**Measured Area density of Ni in the ICI** AD_{Ni} **(ng cm^{-2})**	**Calculated Ni mass**^{d} $M_{tetrapyrrole}$ **(g)**	**Corresponding number of mole for Ni**^{e} n_{Ni} **(mole)**	**Molar concentration of tetrapyrrole**^{f} $C_{tetrapyrrole}$ **(µM)**
**Cell Point 1**	72	14	9	$1.83.10^{-11}$	$2.02.10^{-5}$	278.1	23	$4.65.10^{-14}$	$7.92.10^{-16}$	**43**
**Cell Point 2**	22	13	8	$4.72.10^{-12}$	$5.72.10^{-6}$	205.9	17	$9.72.10^{-15}$	$1.66.10^{-16}$	**35**
**Cell Point 3**	79.3	13.6	8.7	$1.9.10^{-11}$	$2.16.10^{-5}$	224.6	19	$4.10.10^{-14}$	$6.99.10^{-16}$	**37**
**Cell Point 4**	38	13.5	8.7	$9.03.10^{-12}$	$1.03.10^{-5}$	155.6	13	$1.34.10^{-14}$	$2.28.10^{-16}$	**25**

The cells considered are the four cells where the XANES analysis was performed for the *A. tetragonala* from Fig. 3.

$^{a}R_c = 0.64.R_m$; $^{b}V_{arcta} = L .\pi . R_c^2$; $^{c}A_p = L.2. R_m$; $^{d}m_{Ni} = (AD_{Ni})_{ICI} .0.1 . A_p$; $^{e}n_{Ni} = m_{Ni}/M_{Ni}$; $^{f}c_{tetrapyrrole} = n_{Ni}/V_{cell}$;

Supplementary figures

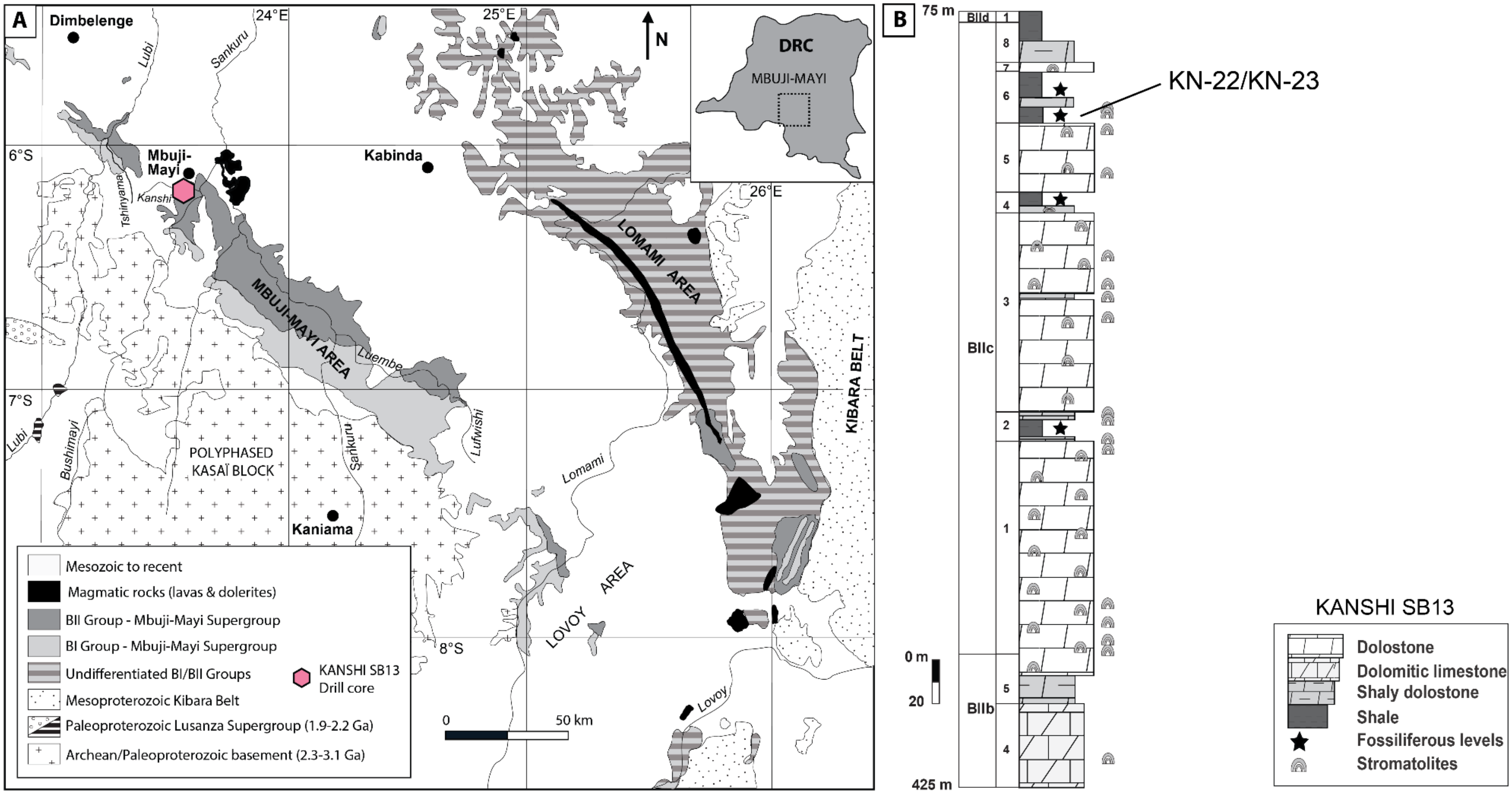

Supplementary Figure 1. **(A)** Geological map of the Sankuru-Mbuji-Mayi-Lomami-Lovoy (SMLL) basin showing the Kanshi SB13 (KN) drill core location and (**B**) Stratigraphy of the Mbuji-Mayi area and location of the samples studied. Modified after (11).

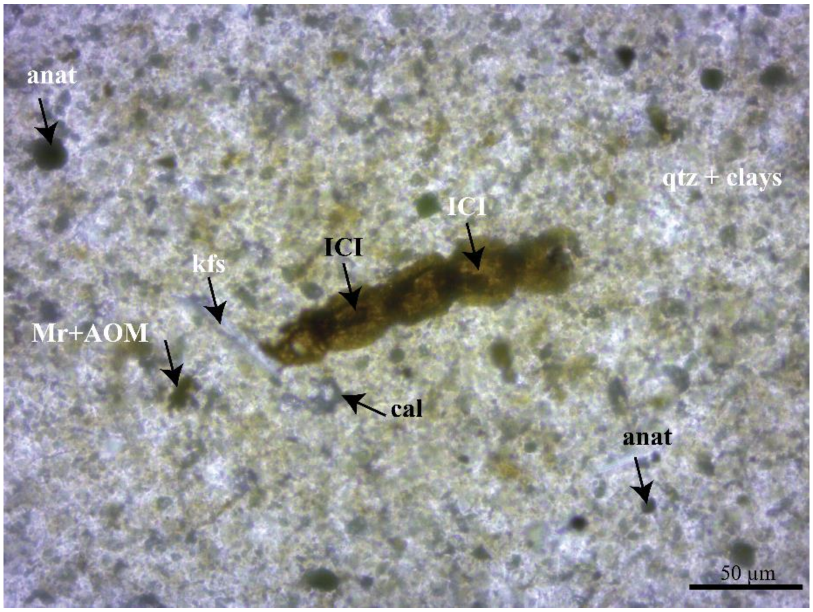

Supplementary Figure 2. Photomicrograph of one embedded specimen of *Arctacellularia tetragonala* in its original matrix. The thin section was cut parallel to the stratification. Anat: anatase, qtz: quartz, Mr: marcasite, AOM: amorphous Organic Matter, kfs: K-felspath, cal: calcite, ICI: intracellular inclusion.

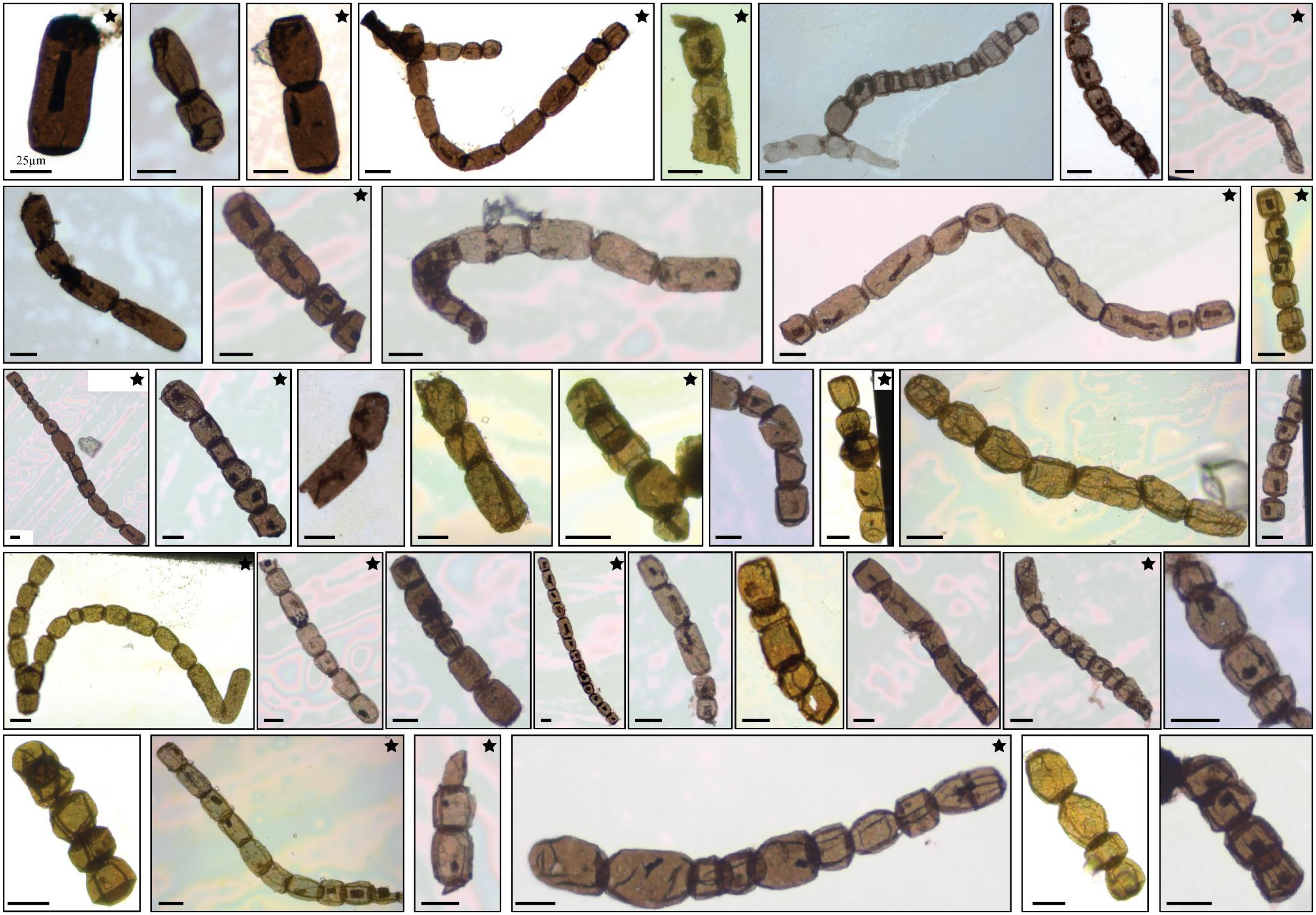

Supplementary Figure 3. Photomicrographs of 37 *Arctacellularia tetragonala* studied in this work showing the diversity in the length and shape of the barrel-shaped cells constituting the uniseriate filaments. Black stars correspond to specimens that have been analysed by SR-µXRF

.

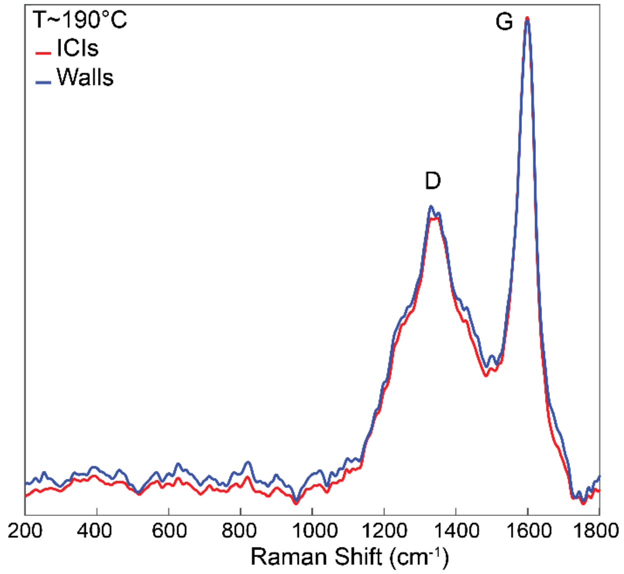

Supplementary Figure 4. Representative Raman spectra of an ICI and the surrounding walls showing a typical feature of poorly ordered carbonaceous material, which experienced a maximum temperature of burial of 190 °C. Spectra and temperatures are comparable to what was obtained on other microfossils from the same sample[6]. These spectra demonstrate the syngenicity of the ICI and the absence of mineral components associated to the ICI, especially sulphides (300-400 cm^{-1} region).

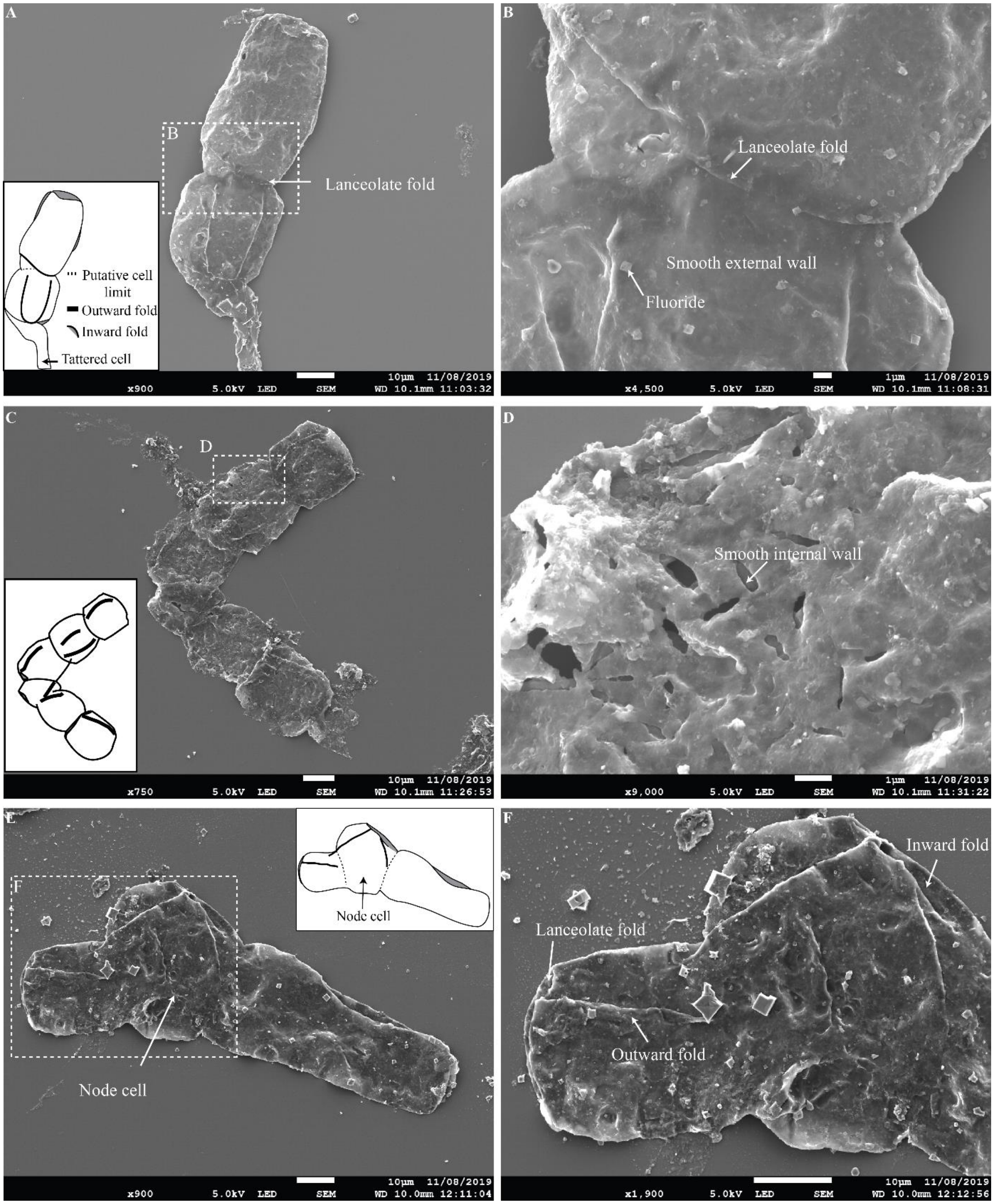

Supplementary Figure 5. SEM images in secondary electron and interpretative sketches showing the smooth unornamented external (**A, B**) and internal walls (**C, D**) and the presence of lanceolate, inward, and outward folds **(E, F**), suggesting a semi-flexible wall.

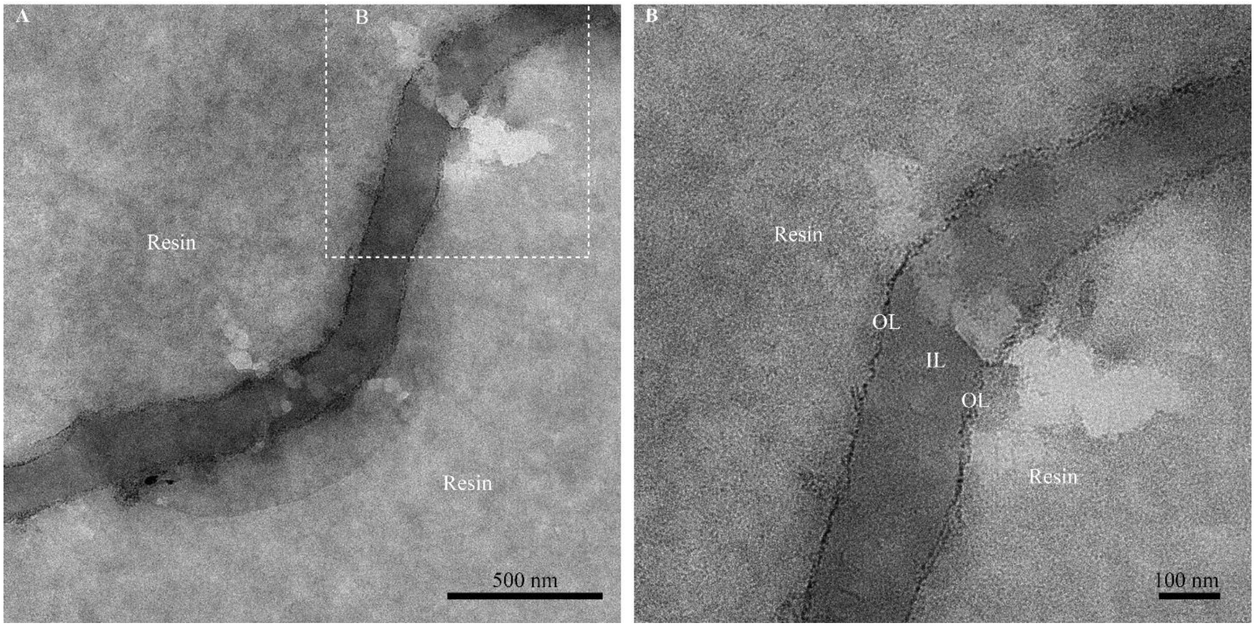

Supplementary Figure 6. TEM images of a compressed *A. tetragonala* cut perpendicularly to the main axis. (**A**) Large view of the section and (**B**) zoom of the dashed box in A showing the bilayered ultrastructure of the wall with an electron-dense outer layer (OL) and an electron-tenuous inner layer (IL).

Supplementary Figure 7. (*Next page*) SR-µXRF imaging of an *A. tetragonala* (Fig. 2A-F) (**A**) Photomicrograph of the investigated microfossil (**B-I**) Fe, Ni, Cu, Zn, S, Ca, K, and Ti SR-µXRF maps obtained in On-the-fly mode at SLS (pixel: 1µm, 200 ms/px). These maps show these elements are mostly homogeneously distributed in the wall. In addition, sulphides (s.) and secondary fluorides (fl.) attached to the surface of the fossil concentrate locally these elements (**J-P**) Fe, Ni, Cu, Zn, S, Ca and K SR-µXRF maps obtained at SS (pixel: 400 nm, 0.1 s/px) of the dashed black squared region. These maps show the specific and homogeneous enrichment of Ni in the ICI compared to wall. Colour scales correspond to normalized counts.

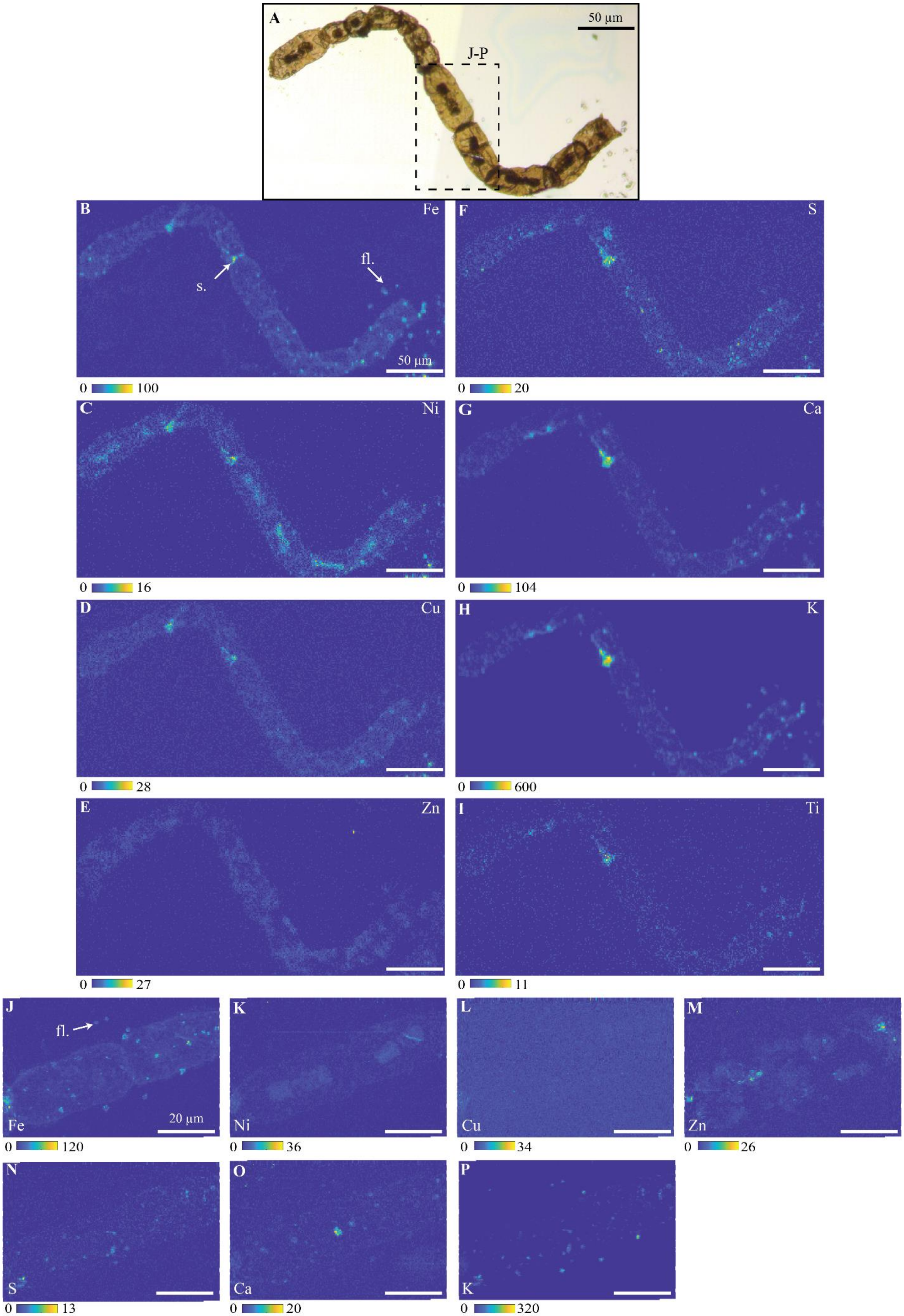
A
50 µm
J-P
B
Fe
s.
fl.
50 µm
0 100
F
S
0 20
C
Ni
0 16
G
Ca
0 104
D
Cu
0 28
H
K
0 600
E
Zn
0 27
I
Ti
0 11
J
fl.
Fe
20 µm
0 120
K
Ni
0 36
L
Cu
0 34
M
Zn
0 26
N
S
0 13
O
Ca
0 20
P
K
0 320

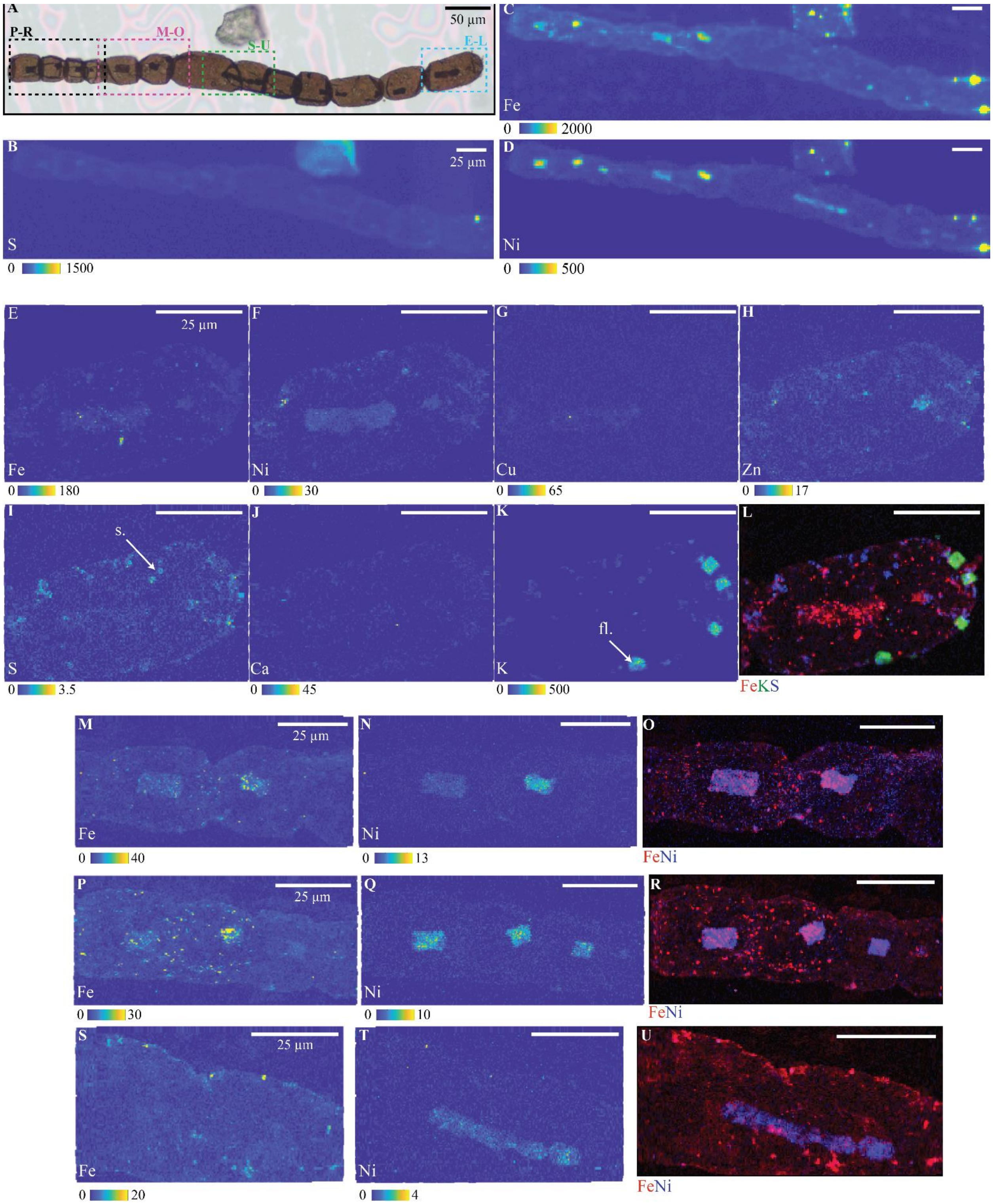
A
50 µm
P-R
M-O
S-U
E-L
C
Fe
0 2000
B
25 µm
S
0 1500
D
Ni
0 500
E
25 µm
Fe
0 180
F
Ni
0 30
G
Cu
0 65
H
Zn
0 17
I
s.
S
0 3.5
J
Ca
0 45
K
fl.
K
0 500
L
FeKS
M
25 µm
Fe
0 40
N
Ni
0 13
O
FeNi
P
25 µm
Fe
0 30
Q
Ni
0 10
R
FeNi
S
25 µm
Fe
0 20
T
Ni
0 4
U
FeNi

Supplementary Figure 8. (*Previous page*) SR-µXRF imaging of an *A. tetragonala* (Fig. 2G-J) (**A**) Photomicrograph of the investigated microfossil (**B-D**) S, Fe & Ni ROI SR-µXRF maps obtained in On-the-fly mode at SLS (pixel: $1 \mu m^2$, 0.2 s/px). (**E-L**) Fe, Ni, Cu, Zn, S, Ca, and K SR-µXRF maps obtained in Stop-and-Go mode at SLS (pixel: $1 \mu m^2$, 1 s/px) and the associated RGB composite image (R: Fe, G: K, B: S) of the blue dashed square in **A**. (**M-O**) Fe and Ni SR-µXRF maps obtained in Stop-and-Go mode at SLS (pixel: $1 \mu m^2$, 1 s/px) and the associated composite image (R: Fe, B: Ni) of the pink dashed square in **A**. (**P-R**) Fe and Ni SR-µXRF maps obtained in Stop-and-Go mode at SLS (pixel: $1 \mu m^2$, 1 s/px) and the associated composite image (R: Fe, B: Ni) of the black dashed square in **A**. (**S-U**) Fe and Ni SR-µXRF maps obtained in Stop-and-Go mode at SLS (pixel: $1 \mu m^2$, 1 s/px) and the associated composite image (R: Fe, B: Ni) of the green dashed square in **A**. All these maps show a specific enrichment of Ni in the ICIs. When Fe is present, it is as hotspots in the ICI. Small sulphides (s.) and fluorides (fl.) are attached to the surface of the fossils. Colour scales correspond to normalized counts.

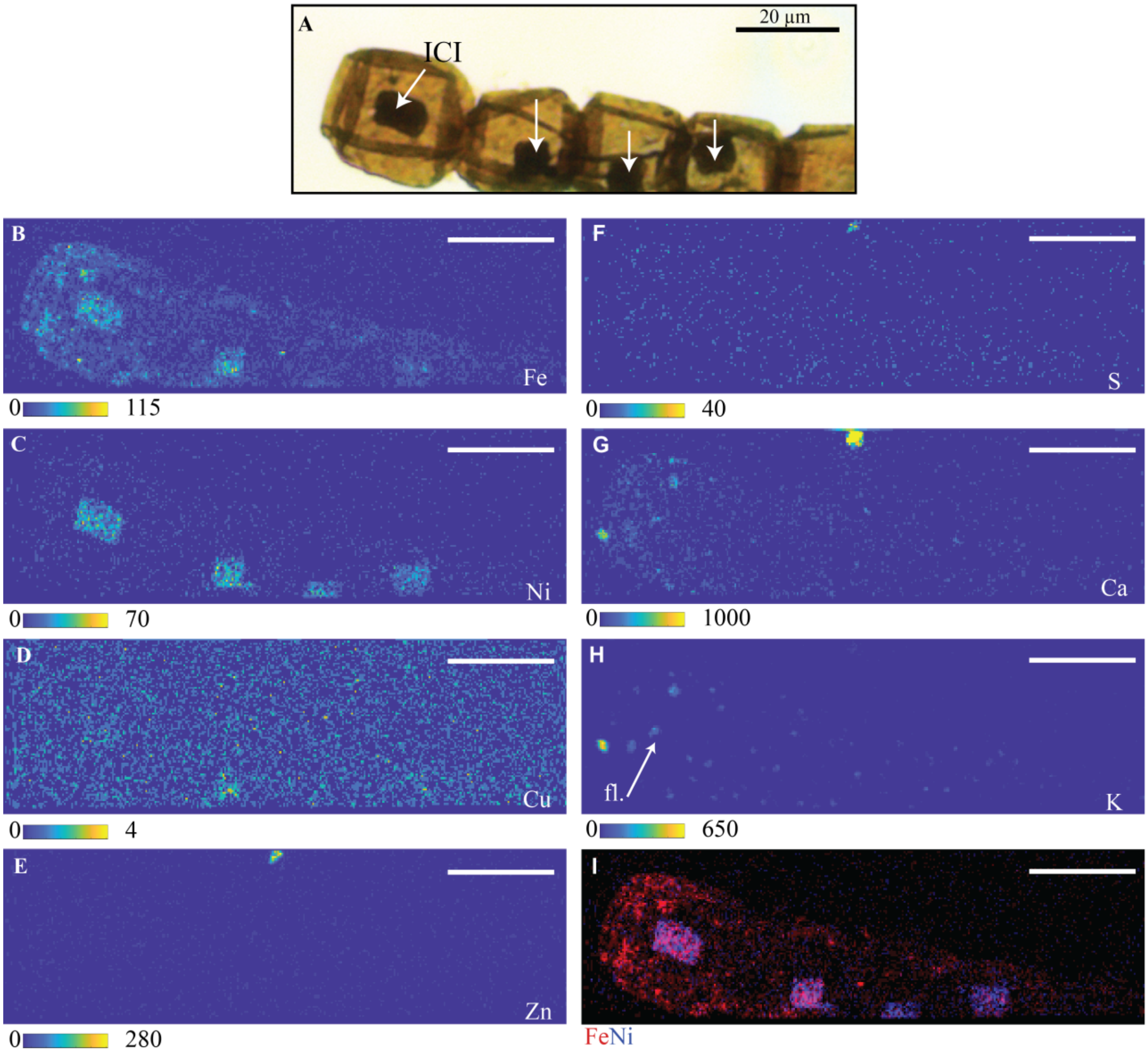

Supplementary Figure 9. SR-µXRF imaging of an *A. tetragonala* showing the specific and homogeneous enrichment of Ni in the ICIs compared to the more heterogeneous distribution of Fe in the ICIs. Ca and K are mainly concentrated in neo-formed fluorides (fl.) (**A**) Photomicrograph of the investigated microfossil. White arrows show the ICIs (**B-I**) Fe, Ni, Cu, Zn, S, Ca, and K SR-µXRF maps obtained at SS (pixel: 350 nm, 0.1 s/px) and the associated composite image (R: Fe, B: Ni). Colour scales correspond to normalized counts.

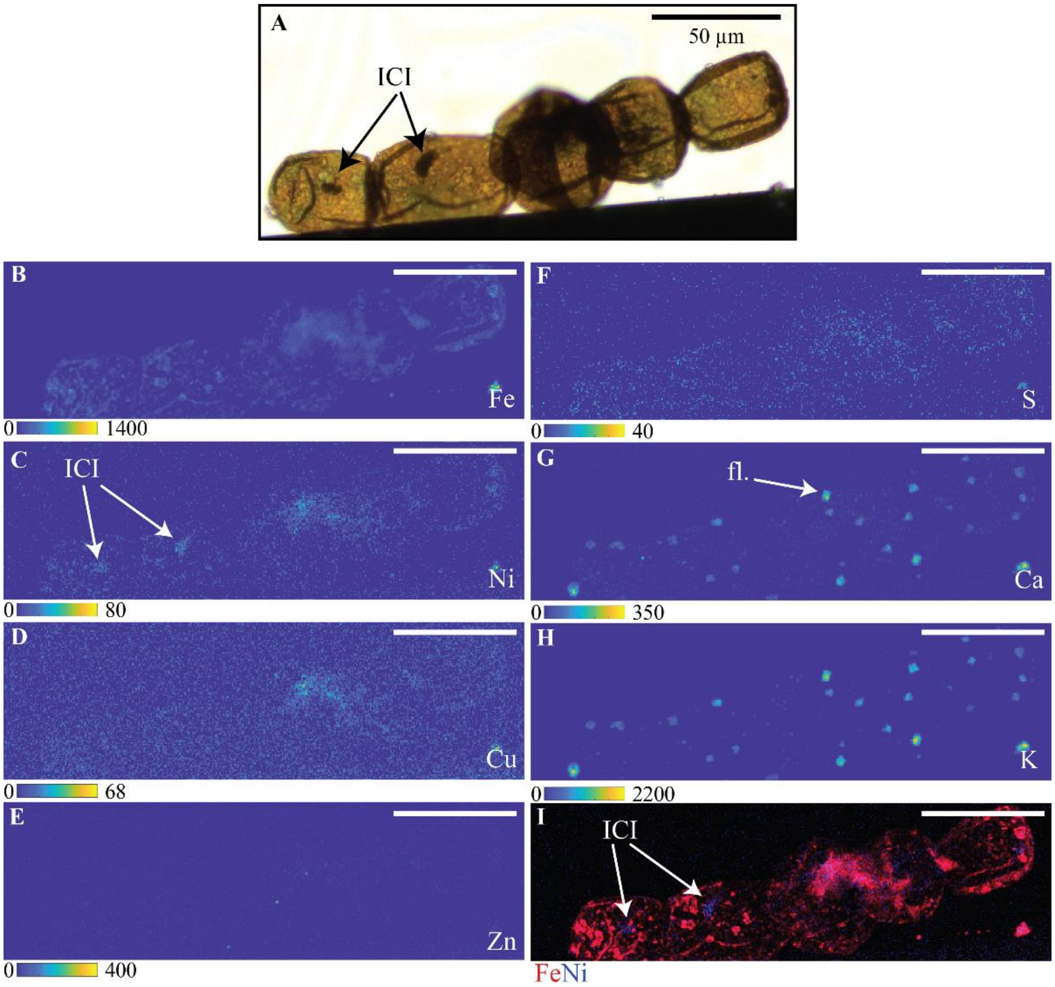

Supplementary Figure 10. SR-µXRF imaging of an *A. tetragonala* showing the specific enrichment of Ni in the ICIs. (**A**) Photomicrograph of the investigated microfossil. Black arrows show the ICIs (**B-I**) Fe, Ni, Cu, Zn, S, Ca, and K SR-µXRF maps obtained at SS (pixel: 400 nm, 0.1 s/px) and the associated RGB composite image (R: Ni, G: Fe, B: Fe). Fe, Ca and K are strongly linked to the fluorides (fl.). White arrows show the position of the two ICI in map **C**. Colour scales correspond to normalized counts.

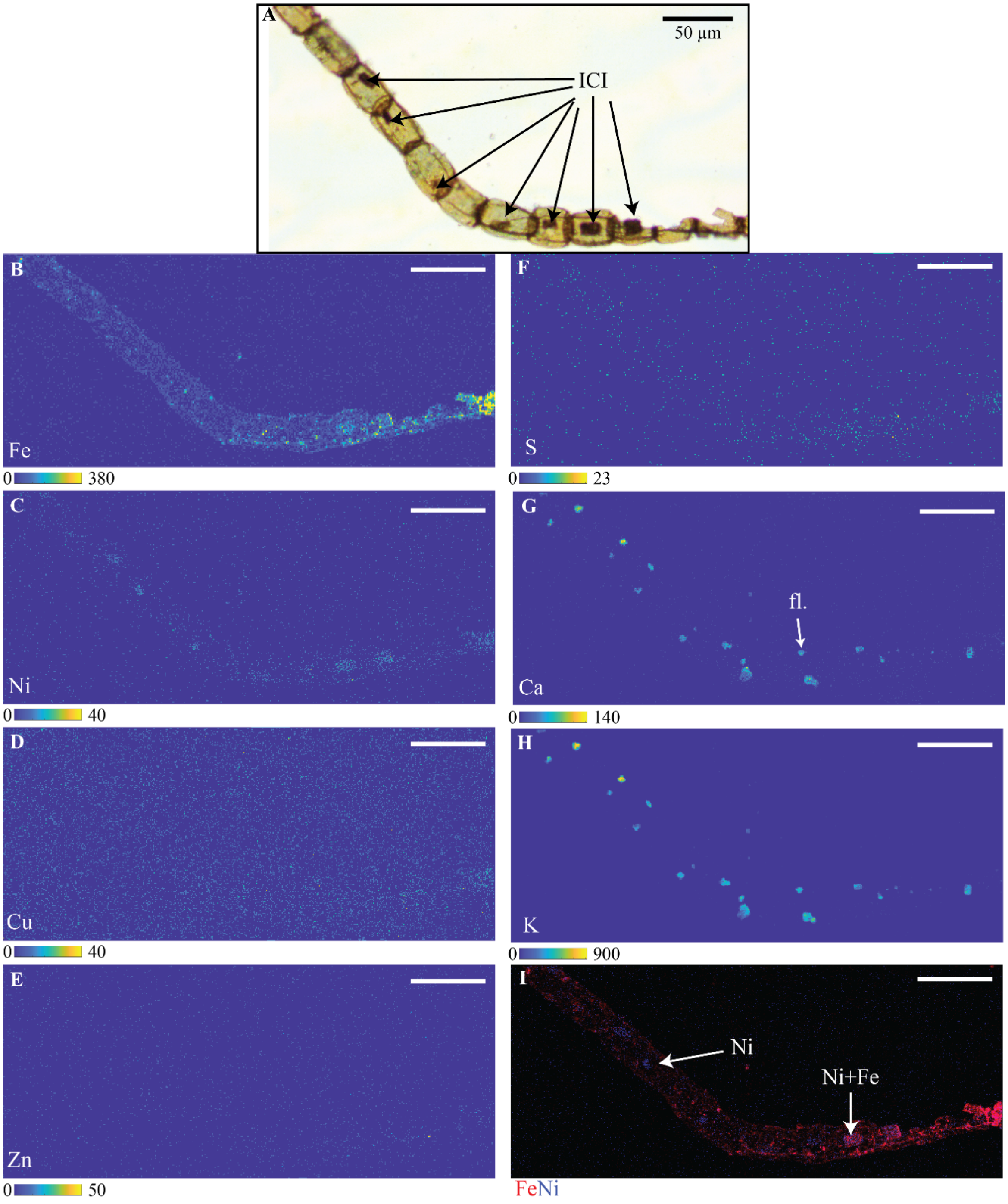

Supplementary Figure 11. SR-µXRF imaging of an *A. tetragonala* showing that along the same fossil ICIs can be enriched in Ni only or in Ni+Fe. The distribution of Ni is homogeneous while Fe is more heterogeneous in the ICIs. (**A**) Photomicrograph of the investigated microfossil. Black arrows show the ICI (**B-I**) Fe, Ni, Cu, Zn, S, Ca, and K SR-µXRF maps obtained at SS (pixel: 400 nm, 0.1 s/px) and the associated composite image (R: Fe, B: Ni). Colour scales correspond to normalized counts.

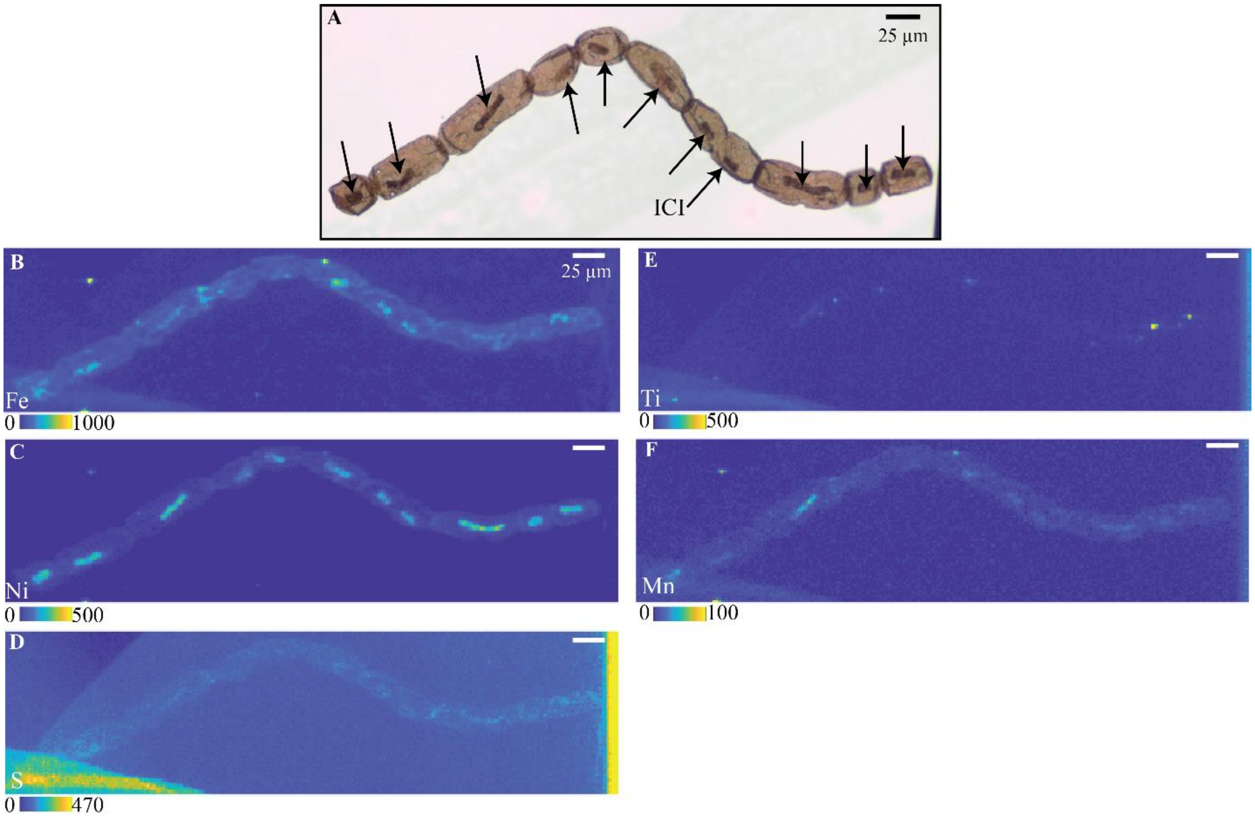

Supplementary Figure 12. SR-µXRF imaging of an *A. tetragonala* (Fig. 3) showing the specific enrichment of Ni in the ICIs. Sometime Fe and Mn are also enriched in the ICIs (**A**) Photomicrograph of the investigated microfossil. Black arrows show the ICI (**B-G**) Fe, Ni, S, Ti, and Mn ROI SR-µXRF maps obtained in On-the-fly mode at SLS (pixel: 1.5 µm, 200 ms/px). Colour scales correspond to normalized counts.

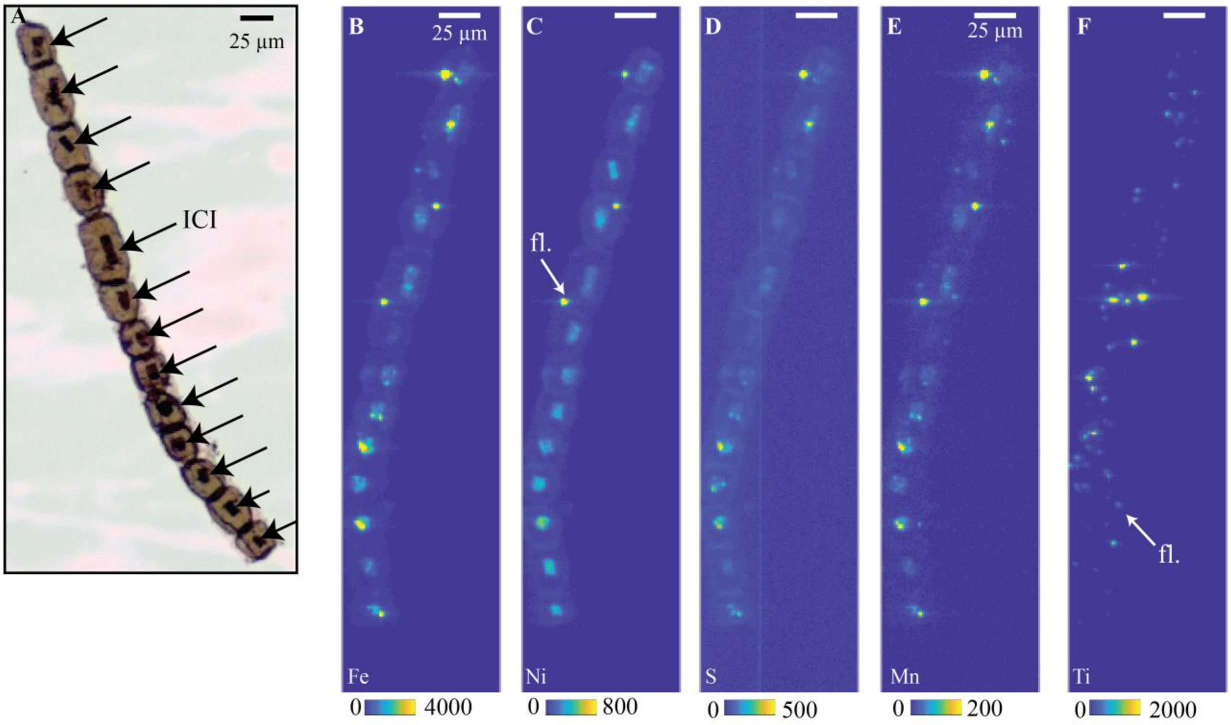

Supplementary Figure 13. SR-µXRF imaging of an *A. tetragonala* showing a specific enrichment of the ICIs by Ni, Fe and S. (**A**) Photomicrograph of the investigated microfossil. Black arrows show the ICIs (**B-F**) Fe, Ni, S, Mn, and Ti ROI SR-µXRF maps obtained in On-the-fly mode at SLS (pixel: 1µm², 200 ms/px). Colour scales correspond to normalized counts. (fl.) is for fluorides.

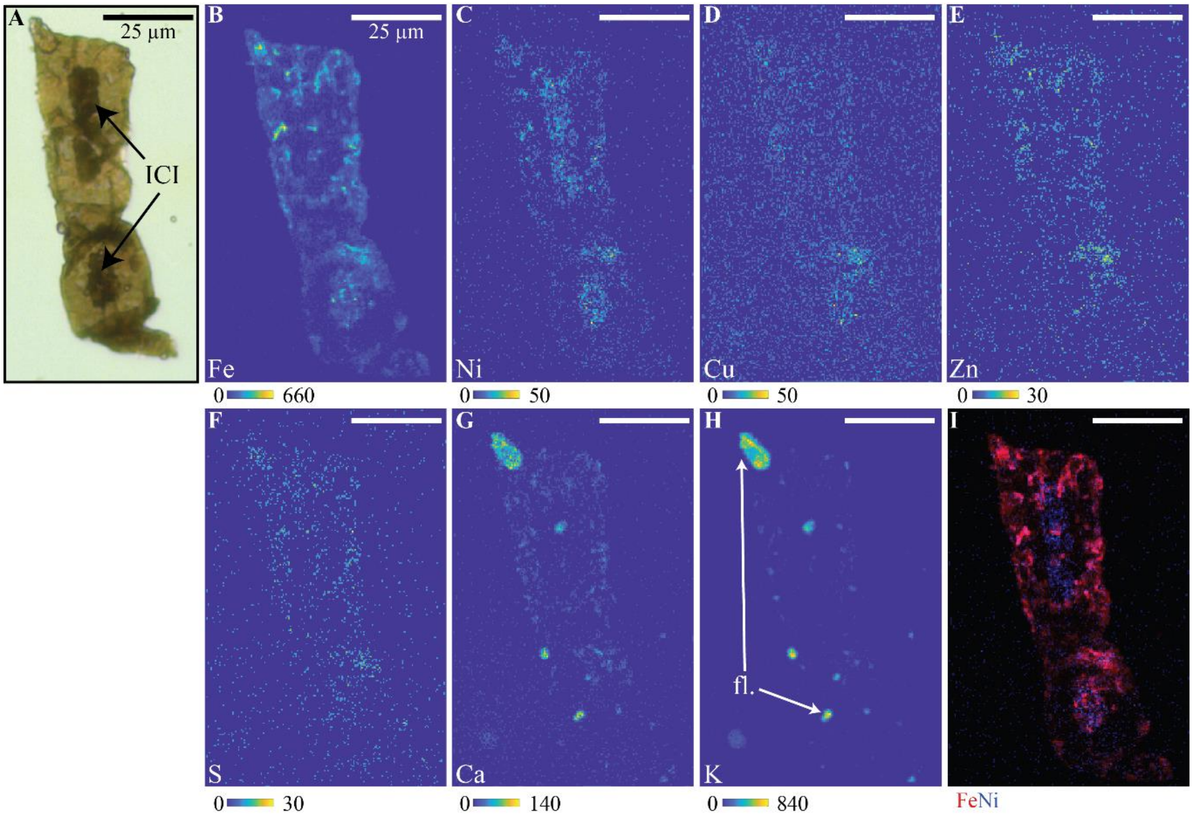

Supplementary Figure 14. SR-µXRF imaging of an *A. tetragonala* showing the specific and homogeneous enrichment of Ni in the two ICIs compared to the heterogeneous distribution of Fe in the bottom ICI. Fe is putatively associated to small mineral phases. (**A**) Photomicrograph of the investigated microfossil. Black arrows show the ICIs (**B-H**) Fe, Ni, Cu, Zn, S, Ca, and K SR-µXRF maps obtained at SS (pixel: 400 nm, 0.1 s/px). The metals and S are distributed more or less homogeneously in the walls while Ca and K are concentrated in fluorides (fl.). (**I**) associated composite image (R: Fe, B: Ni). Colour scales correspond to normalized counts.

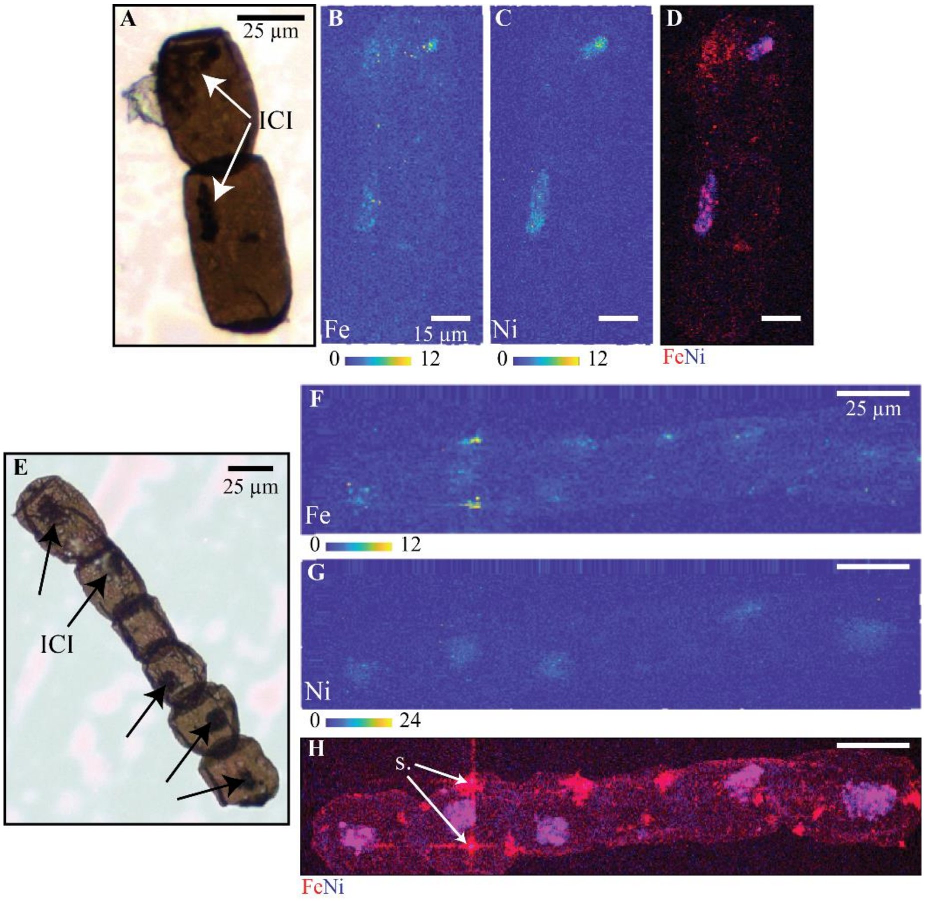

Supplementary Figure 15. SR-µXRF imaging of two specimens of *A. tetragonala* showing an enrichment of Ni in their ICIs. Fe is present in the ICIs as small hotspots. (**A, E**) Photomicrograph of the two investigated microfossils. White and black arrows show the ICIs (**B-D**) Fe and Ni SR-µXRF maps obtained at SS (pixel: 400 nm, 0.1 s/px) and the associated composite image (R: Fe, B: Ni). The maps for other elements are not showed as their counts were very low. Scale bar is 15 µm for all the maps. (**F-H**) Fe and Ni SR-µXRF maps obtained at SS (pixel: 400 nm, 0.1 s/px) and the associated composite image (R: Fe, B: Ni). Again the other elements are not showed due to their low counts. (s.) is for sulphides attached to the surface of the fossil. Colour scales correspond to normalized counts.

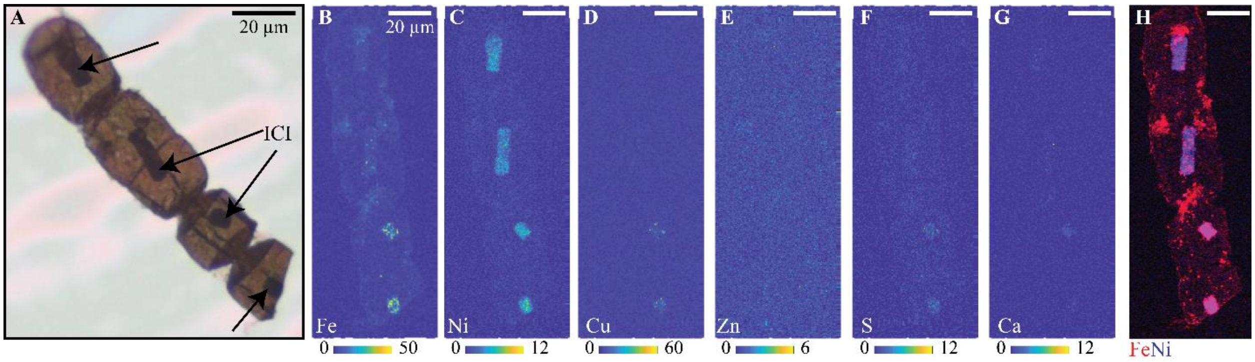

Supplementary Figure 16. SR-µXRF imaging of an *A. tetragonala* showing that depending on the cell in the fossil, the ICIs are specifically enriched either only in Ni or in Ni and Fe. When Fe is present it forms small hotspots within the ICI (**A**) Photomicrograph of the investigated microfossil. Black arrows show the ICI (**B-H**) Fe, Ni, Cu, Zn, S, and Ca SR-µXRF maps obtained at SS (pixel: 400 nm, 0.15 s/px) and the associated composite image (R: Fe, B: Ni). Colour scales correspond to normalized counts.

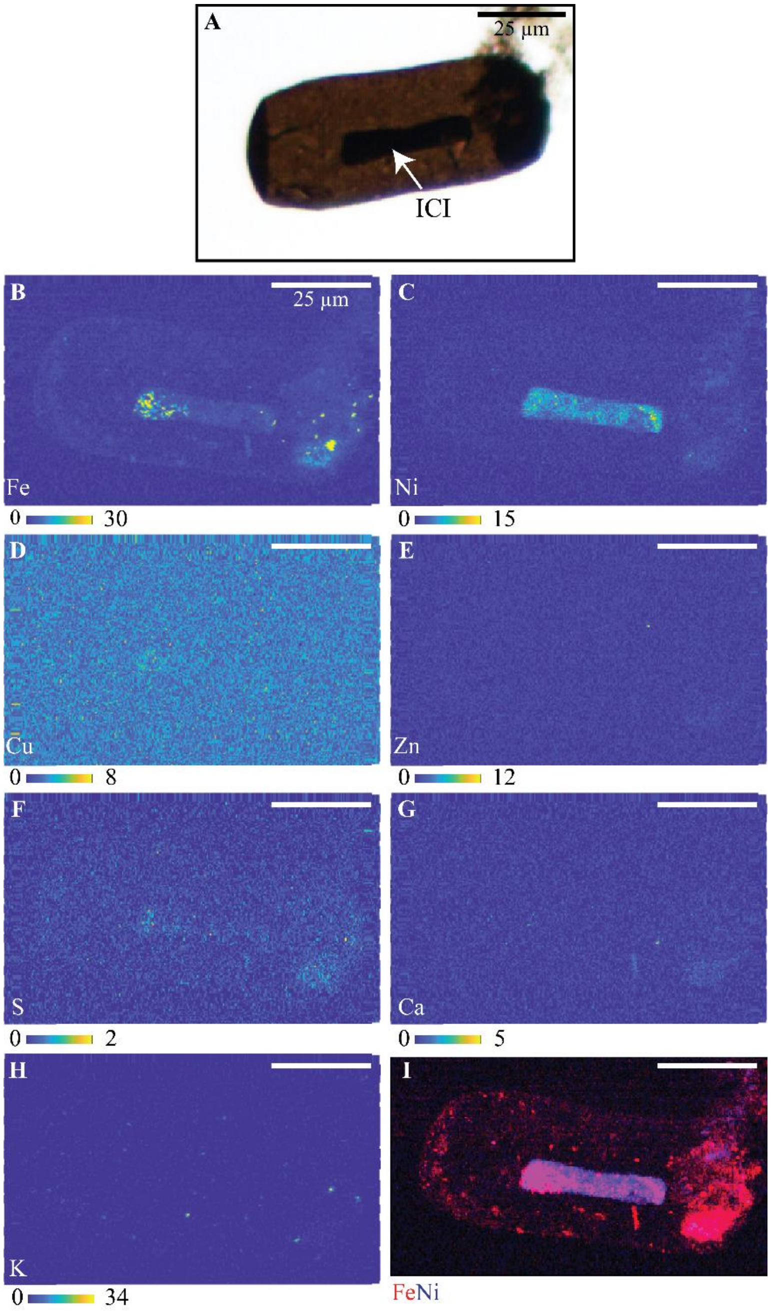

Supplementary Figure 17. SR-µXRF imaging of an *A. tetragonala* showing a homogeneous enrichment of Ni in the ICI and small Fe hotspots in the left part of the ICI. (**A**) Photomicrograph of the investigated microfossil. White arrow shows the ICI (**B-I**) Fe, Ni, Cu, Zn, S, Ca, and K SR-µXRF maps obtained at SS (pixel: 350 nm, 0.15 s/px) and the associated RGB composite image (R: Fe, G: Ni, B: Ni). Colour scales correspond to normalized counts.

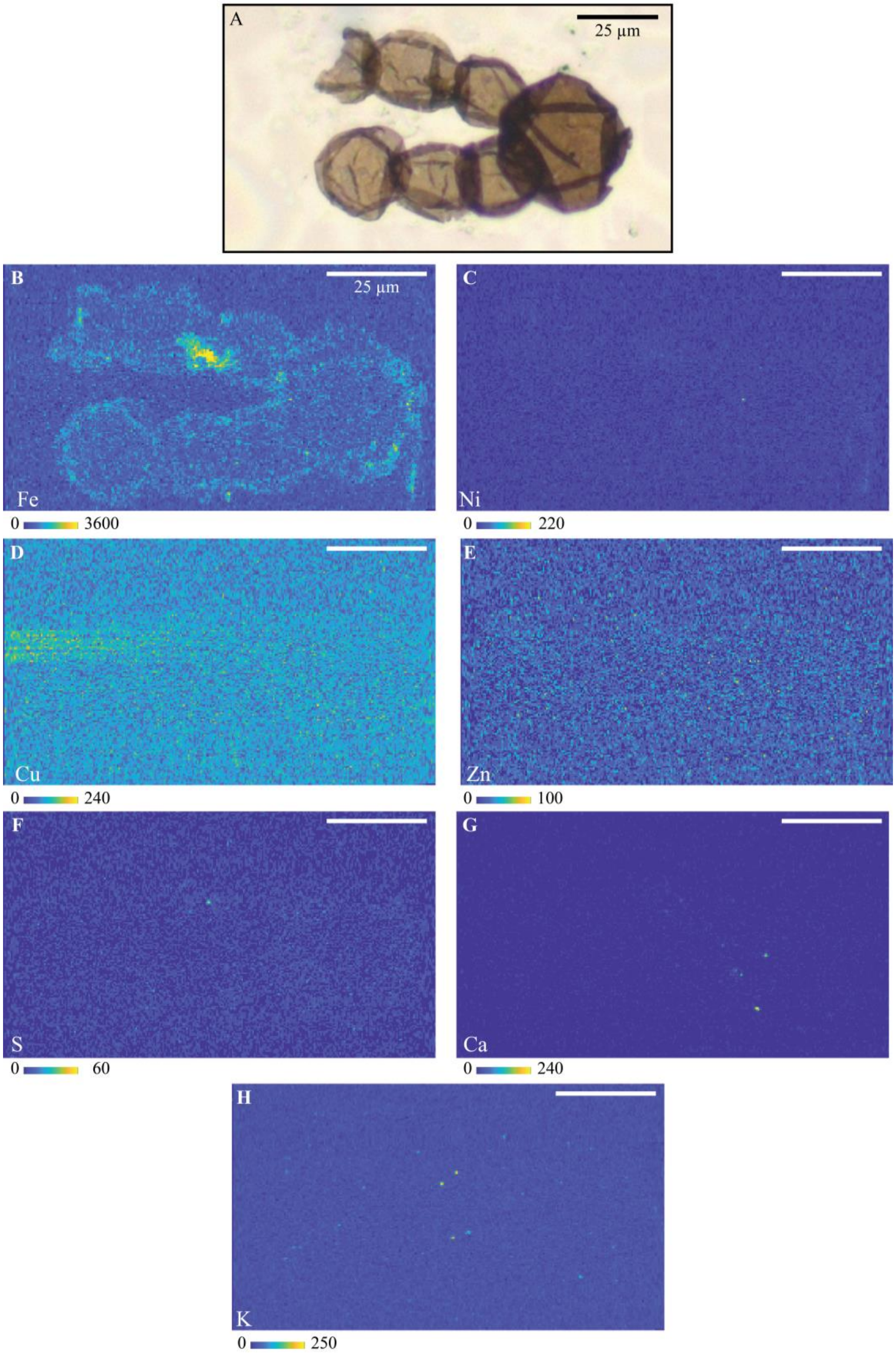

Supplementary Figure 18. SR-µXRF imaging of an *A. tetragonala* without ICI showing that the wall is only enriched in Fe. (**A**) Photomicrograph of the investigated microfossil. (**B-H**) Fe, Ni, Cu, Zn, S, Ca, and K SR-µXRF maps obtained at SS (pixel: 400 nm, 0.1 s/px). Colour scales correspond to normalized counts.

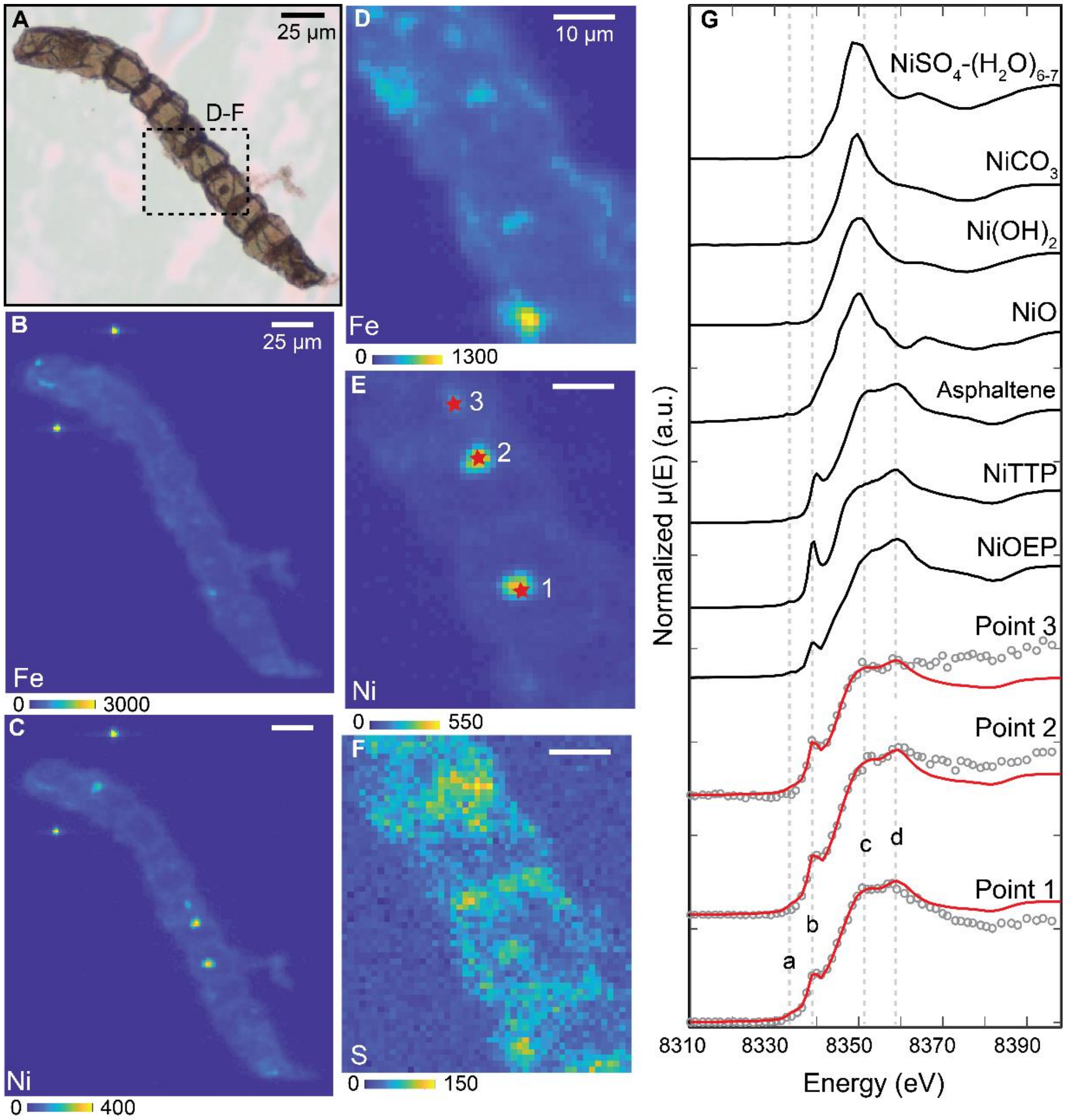

Supplementary Figure 19. Presence of bound porphyrinic species highlighted by XANES analyses on three ICIs. (**A**) Photomicrograph of the investigated microfossil. (**B-C**) Fe and Ni SR-µXRF maps obtained in On-the-Fly mode at SLS (pixel: 1µm, 200 ms/px) showing the enrichment in Ni of the ICIs. (**D-F**) Fe, Ni and S SR-µXRF maps obtained in Stop-and-Go mode at SLS (pixel: 1µm, 1 s/px) showing the enrichment in Ni of the ICIs. (**G**) XANES spectra at the Ni K-edge of 3 ICIs performed in zones with low Fe content (grey circles), their linear combination fitting (red lines) and XANES spectra of two Ni-porphyrin standards (NiTPP: Ni-tetraphenylporphyne; NiOEP: Ni-octaethylporphyne)[43], asphaltene[44], NiO, $Ni(OH)_2$, $NiCO_3$ and $NiSO_4$-$(H2O)_{6-7}$ standards. The shoulder and the spectral line shape are typical of Ni

in coordination (IV) in Ni-porphyrins incorporated in kerogen. Differences between the fitted spectra and the data comes from the molecular heterogeneities between the standards used for the fitting and the incorporated tetrapyrroles moieities in the kerogen. Colour scales correspond to normalized counts. See main text for signification a, b, c and d.

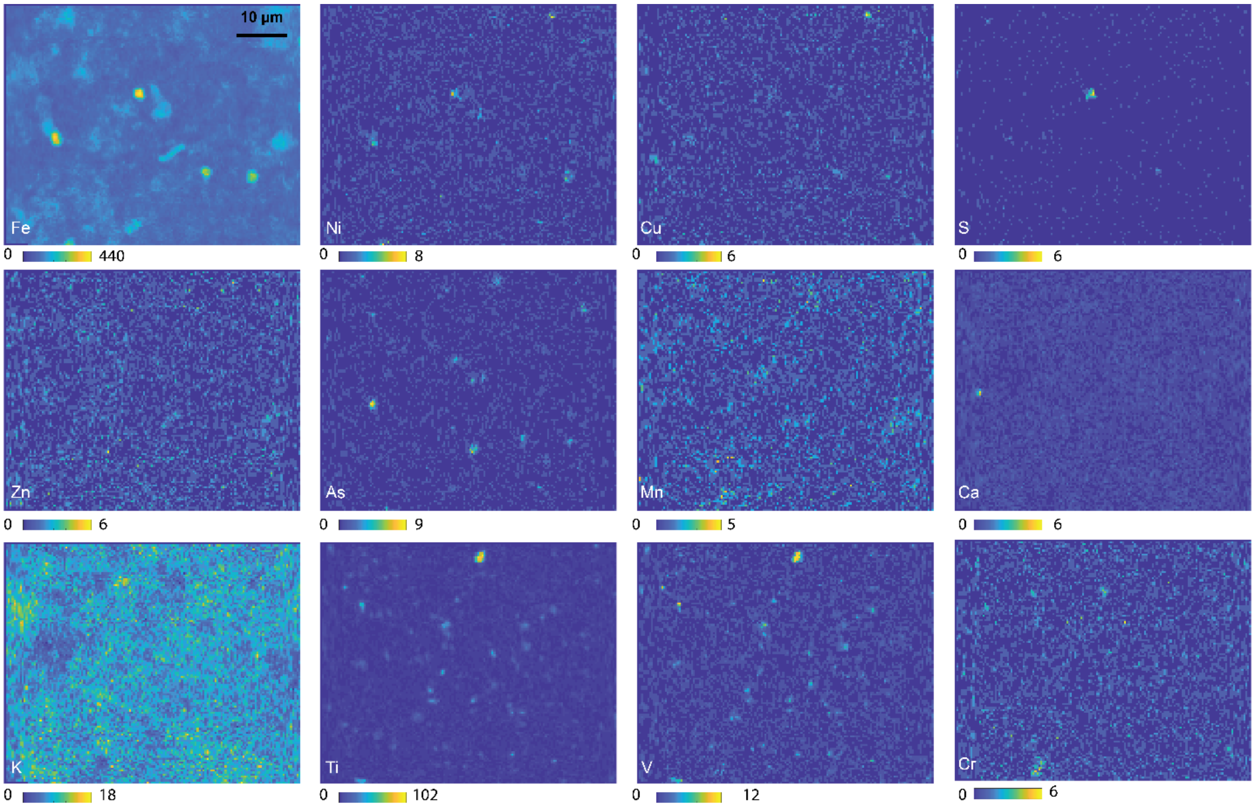

Supplementary Figure 20. SR-µXRF imaging of the shales from which the investigated microfossil are retrieved showing that the matrix is principally constituted of clays with rare K-feldspath, anatase and marcasite.

Additional References

1. Kadima, E., Delvaux, D., Sebagenzi, S. N., Tack, L. & Kabeya, S. M. Structure and geological history of the Congo Basin: An integrated interpretation of gravity, magnetic and reflection seismic data. *Basin Res.* **23**, 499–527 (2011).
2. Kadima Kabongo, É., Ntabwoba, S. S. M. & Lucazeau, F. A Proterozoic-rift origin for the structure and the evolution of the cratonic Congo basin. *Earth Planet. Sci. Lett.* **304**, 240–250 (2011).
3. Delvaux, D. & Fernando-Alonso, M. Petroleum Potential of the Congo Basin. in *Geology and Resource Potential of the Congo Basin. Regional Geology Reviews* (eds. de Wit, M. J., Guillocheau, F. & de Wit, M. C. J.) 371–391 (Springer, 2015).
4. Delpomdor, F., Blanpied, C., Virgone, A. & Préat, A. Sedimentology and Sequence Stratigraphy of the Late Precambrian Carbonates of the Mbuji-Mayi Supergroup in the Sankuru-Mbuji-Mayi-Lomami-Lovoy Basin (Democratic Republic of the Congo). in *Geology and Resource Potential of the Congo Basin* (eds. De Wit, M. J., Guillocheau, F. & De Wit, M. C. J.) 59–76 (Springer-Verlag Berlin Heidelberg, 2015).
5. Raucq, P. Contribution a la connaissance du systeme de la Bushimay (Congo Belge). *Ann. du Musée R. du Congo Belge -Sciences Géologiques* **18**, (1957).
6. Baludikay, B. K. et al. Raman microspectroscopy, bitumen reflectance and illite crystallinity scale: comparison of different geothermometry methods on fossiliferous Proterozoic sedimentary basins (DR Congo, Mauritania and Australia). *Int. J. Coal Geol.* **191**, 80–94 (2018).
7. Wazilewski, I. Exploration en profondeur des formations du Système de la Bushimay (Bakwanga, Kasai, Congo Belge). (Univiversité de Louvain, 1954).
8. Raucq, P. Nouvelles acquisitions sur filesysteme de la Bushimay. *Ann. du Mus. l'Afrique Cent. Sci. Geol.* **8**, 69 (1970).
9. Delpomdor, F., Blanpied, C., Virgone, A. & Préat, A. Paleoenvironments in Meso-Neoproterozoic carbonates of the Mbuji-Mayi Supergroup (Democratic Republic of Congo) - Microfacies analysis

combined with C-O-Sr isotopes, major-trace elements and REE+Y distributions. *J. African Earth Sci.* **88**, 72–100 (2013).

10. Delpomdor, F. et al. Depositional age, provenance, and tectonic and paleoclimatic settings of the late mesoproterozoic-middle neoproterozoic Mbuji-Mayi Supergroup, Democratic Republic of Congo. *Palaeogeogr. Palaeoclimatol. Palaeoecol.* **389**, 4–34 (2013).

11. François, C. et al. Contributions of U-Th-Pb dating on the diagenesis and sediment sources of the lower group (BI) of the Mbuji-Mayi Supergroup (Democratic Republic of Congo). *Precambrian Res.* **298**, 202–219 (2017).

12. Cahen, L., Ledent, D. & Snelling, N. J. *Données Geochronologiques dans le Katangien inferieur du Kasai oriental et du Shaba nord-oriental (Republique du Zaire).* (1974).

13. Cahen, L., Delhal, J., Vail, J. R., Bonhomme, M. & Ledent, D. The Congo (Zaire) basin and the Kasai and north-eastern Angola Shield. in *The Geochronology and Evolution of Equatorial Africa* (1984).

14. Delpomdor, F. & Préat, A. Early and late Neoproterozoic C, O and Sr isotope chemostratigraphy in the carbonates of West Congo and Mbuji-Mayi Supergroups: A preserved marine signature? *Palaeogeogr. Palaeoclimatol. Palaeoecol.* **389**, 35–47 (2013).

15. Baludikay, B. K., Storme, J. Y., François, C., Baudet, D. & Javaux, E. J. A diverse and exquisitely preserved organic-walled microfossil assemblage from the Meso-Neoproterozoic Mbuji-Mayi Supergroup (Democratic Republic of Congo) and implications for Proterozoic biostratigraphy. *Precambrian Res.* **281**, 166–184 (2016).

16. Baludikay, B. K. Biostratigraphie, paléoécologie et évolution thermique du Supergroupe Mésoprotérozoïque de Mbuji-Mayi, RdCongo. (PhD thesis, Université de Liège, 2018).

17. Hermann, T. N. & Podkovyrov, V. N. On the nature of the Precambrian microfossils Arctacellularia and Glomovertella. *Paleontol. J.* **42**, 655–664 (2008).

18. Fehling, J., Stoecker, D. & Baldauf, S. L. Photosynthesis and the Eukaryote Tree of Life. in *Evolution of Primary Producers in the Sea* 75–107 (Elsevier Inc., 2007).

19. Butterfield, N. J. A vaucheriacean alga from the middle Neoproterozoic of Spitsbergen: implications for the evolution of Proterozoic eukaryotes and the Cambrian explosion. *Paleobiology* **30**, 231–252 (2004).

20. Graham, L. E. & Wilcox, L. W. *Algae*. (Prentice Hall, 2000).

21. Shukla, Y., Sharma, M. & Sergeev, V. N. Organic walled microfossils from the Neoproterozoic Owk Shale, Kurnool Group, South India. *Palaeoworld* (2019).

22. Crawford, A. R. & Compston, W. The age of the Cuddapah and Kurnool systems, southern India. *J. Geol. Soc. Aust.* **19**, 453–464 (1973).

23. Raman, P. K. & Murty, V. N. *Geology of Andhra Pradesh*. (Geological Society of India, 1997).

24. Xing, Y., Duan, C., Liang, Y. & Cao, R. Late Precambrian Palaeontology of China. *Geol. Mem*. **2**, 1–243 (1985).

25. Zhang, S. H., Zhao, Y., Ye, H. & Hu, G. H. Early Neoproterozoic emplacement of the diabase sill swarms in the Liaodong Peninsula and pre-magmatic uplift of the southeastern North China Craton. *Precambrian Res.* **272**, 203–225 (2016).

26. Li, G., Pang, K., Chen, L., Zhou, G., Han, C., Yang, L., Wang, W., Yang, F. & Yin, L. Organic-walled microfossils from the Tonian Tongjiazhuang Formation of the Tumen Group in western Shandong, North China Craton and their biostratigraphic significance. *Gondwana Res.* **76**, 260–289 (2019).

27. Han, C. M., Chen, L., Li, G.-J., Pang, K., Wang, W., Zhou, G.-Z., Yang, L., Lyu, W.-G., Wang, K., Zhong, Z.-H., Wu, C.-X & Yang, F.-J. First record of organic-walled microfossils from the Tonian Shiwangzhuang Formation of the Tumen Group in western Shandong, North China. *Palaeoworld* **30**, 208–219 (2021).

28. Timofeev, B. V., Hermann, T. N. & Mikhailova, N. S. *Microphytofossils of the Precambrian, Cambrian and Ordovician.* (Nauka, 1976).

29. Hermann, T. N. *Organic World Billion Year Ago*. (Nauka, 1990).

30. Jankauskas, T. V. *Microfossils of the Riphean of the South Urals, the Riphean Stratotype, Paleontology, Paleomagnitism.* (Akademia Nauk SSSR, 1982).

31. Jankauskas, T. V., Mikhailova, N. S. & Hermann, T. N. *Precambrian Microfossils of the USSR*. (Nauka, 1989).

32. Zaitseva, T. S. et al. Mössbauer characteristics, mineralogy and isotopic age (Rb-Sr, K-Ar) of Upper Riphean glauconites from the UK Formation, the southern Urals. *Stratigr. Geol. Correl.* **16**, 227–247 (2008).

33. Couëffé, R. & Vecoli, M. New sedimentological and biostratigraphic data in the Kwahu Group (Meso- to Neo-Proterozoic), southern margin of the Volta Basin, Ghana: Stratigraphic constraints and implications on regional lithostratigraphic correlations. *Precambrian Res.* **189**, 155–175 (2011).

34. Kalsbeek, F., Frei, D. & Affaton, P. Constraints on provenance, stratigraphic correlation and structural context of the Volta basin, Ghana, from detrital zircon geochronology: An Amazonian connection? *Sediment. Geol.* **212**, 86–95 (2008).

35. Hofmann, H. J. & Jackson, G. D. Shale-Facies Microfossils from the Proterozoic Bylot Supergroup, Baffin Island, Canada. *J. Paleontol.* **37**, 1–39 (1994).

36. Gibson, T. M. et al. Precise age of Bangiomorpha pubescens dates the origin of eukaryotic photosynthesis. *Geology* **46**, 6–9 (2017).

37. Loron, C. C., Rainbird, R. H., Turner, E. C., Greenman, J. W. & Javaux, E. J. Organic-walled microfossils from the late Mesoproterozoic to early Neoproterozoic lower Shaler Supergroup (Arctic Canada): Diversity and biostratigraphic significance. *Precambrian Res.* **321**, 349–374 (2019).

38. Van Acken, D., Thomson, D., Rainbird, R. H. & Creaser, R. A. Constraining the depositional history of the Neoproterozoic Shaler Supergroup, Amundsen Basin, NW Canada: Rhenium-osmium dating of black shales from the Wynniatt and Boot Inlet Formations. *Precambrian Res.* **236**, 124–131 (2013).

39. Rayner, N. M. & Rainbird, R. H. U-Pb Geochronology of the Shaler Supergroup , Victoria Island, northwest Canada : 2009-2013. *Geol. Surv. Canada, Open File 7419* 62p (2013).

40. Maithy, P. K. Micro-organisms from the Bushimay System (Late Precambrian) of Kanshi, Zaire. *The Palaebotanist* **22**, 133–149 (1975).

41. Beghin, J., Storme, J.-Y., Blanpied, C., Gueneli, N., Brocks, J.J., Poulton, S.W. & Javaux, E.J. Microfossils from the late Mesoproterozoic – early Neoproterozoic Atar/El Mreïti Group, Taoudeni Basin, Mauritania, northwestern Africa. *Precambrian Res.* **291**, 63–82 (2017).

42. Rooney, A. D., Selby, D., Houzay, J. P. & Renne, P. R. Re-Os geochronology of a Mesoproterozoic sedimentary succession, Taoudeni basin, Mauritania: Implications for basin-wide correlations and Re-Os organic-rich sediments systematics. *Earth Planet. Sci. Lett.* **289**, 486–496 (2010).

43. Nesbitt, J. A., Lindsay, M. B. J. J. & Chen, N. Geochemical characteristics of oil sands fluid petroleum coke. *Appl. Geochemistry* **76**, 148–158 (2017).

44. Lytle, F. W. Cold Lake Asphaltene V and Ni XAS Spectra. *International X-ray Absorption Society XAFS Database.* (1983).
